# Immunological predictors of CD4^+ ^T cell decline in antiretroviral treatment interruptions

**DOI:** 10.1186/1471-2334-8-20

**Published:** 2008-02-26

**Authors:** Elena Seoane, Salvador Resino, Santiago Moreno, Juan Carlos Lopez Bernaldo de Quiros, Ana Moreno, Rafael Rubio, Juan Gonzalez-García, José Ramón Arribas, Federico Pulido, Ma Ángeles Muñoz-Fernández

**Affiliations:** 1 Laboratorio de Inmuno-Biología Molecular, Hospital General Universitario "Gregorio Marañón", Madrid, Spain; 2 Unidad de Investigación, Instituto de Salud Carlos III, Majadahonda, Madrid, Spain; 3 Enfermedades Infecciosas; Hospital Universitario "Ramón y Cajal", Madrid, Spain; 4 Enfermedades Infecciosas/VIH; Hospital Universitario "Gregorio Marañón". Madrid, Spain; 5 Unidad de Infección VIH; Hospital Universitario "12 de Octubre", Madrid, Spain; 6 Enfermedades Infecciosas; Hospital Universitario "La Paz". Madrid, Spain

## Abstract

**Background:**

The common response to stopping anti-HIV treatment is an increase of HIV-RNA load and decrease in CD4^+^, but not all the patients have similar responses to this therapeutic strategy. The aim was to identify predictive markers of CD4^+ ^cell count declines to < 350/μL in CD4-guided antiretroviral treatment interruptions.

**Methods:**

27 HIV-infected patients participated in a prospective multicenter study in with a 24 month follow-up. Patients on stable highly active antiretroviral therapy (HAART), with CD4^+ ^count > 600/μL, and HIV-RNA < 50 copies/ml for at least 6 months were offered the option to discontinue antiretroviral therapy. The main outcome was a decline in CD4^+ ^cell count to < 350/μL.

**Results:**

After 24 months of follow-up, 16 of 27 (59%) patients (who discontinued therapy) experienced declines in CD4^+ ^cell count to < 350/μL. Patients with a CD4^+ ^nadir of < 200 cells/μL had a greater risk of restarting therapy during the follow-up (RR (CI95%): 3.37 (1.07; 10.36)). Interestingly, lymphoproliferative responses to *Mycobacterium tuberculosis *purified protein derivative (PPD) below 10000 c.p.m. at baseline (4.77 (1.07; 21.12)), IL-4 production above 100 pg/mL at baseline (5.95 (1.76; 20.07)) in PBMC cultured with PPD, and increased IL-4 production of PBMC with p24 antigen at baseline (1.25 (1.01; 1.55)) were associated to declines in CD4^+ ^cell count to < 350/μL.

**Conclusion:**

Both the number (CD4^+ ^nadir) and the functional activity of CD4^+ ^(lymphoproliferative response to PPD) predict the CD4^+ ^decrease associated with discontinuation of ART in patients with controlled viremia.

## Background

After more than ten years of use, combination antiretroviral therapy (ART) continues to have a significant impact on the morbidity and mortality of HIV infection [1,2]. Despite significant improvement in the toxicity profile and the convenience of administration of currently recommended therapies, the need for lifelong ART makes it desirable to find ways of decreasing the exposure to drugs [3]. Treatment interruption has been proposed as one such a possibility and it has been explored in several clinical studies in different contexts [4-6].

Recently, large clinical trials have concluded that treatment interruptions can no longer be recommended due to the risk of clinical progression in some circumstances [4]. However, other studies, both retrospective and prospective, carried out under different conditions, have reached different conclusions [5,6]. In these studies, patients who discontinue therapy are not at an increased risk of clinical progression provided that ART is reinitiated before CD4^+ ^counts fall below 200 cells/mm^3^. These studies have uniformly identified the nadir CD4^+ ^count or the CD4^+ ^count at the time of initiation of ART as the single, most important predictive marker of the duration of treatment interruption [4-6]. No other host factors have been identified so far, and in particular, there is no data on the relevance of the functional activity of CD4^+ ^T-cells in the decrease of CD4^+ ^counts in persons who discontinue successful ART. The aim of this study was to identify predictive markers for declines in CD4^+ ^cell count to < 350/μL and to evaluate the long-term effects of CD4-guided antiretroviral treatment interruptions (TI).

## Methods

### Population and study design

A prospective study of the immune, viral, and clinical outcomes of ART interruptions in 27 patients with chronically suppressed HIV-1 infection was carried out in four Spanish hospitals to evaluate the long-term effects of ART interruptions. In addition to the main results of the study, we report herein the factors that have been identified as predictors of the likelihood of a CD4^+ ^count decrease after a follow-up of 24 months, in these 27 patients.

The decision to interrupt ART was made jointly by the physician and the patient, during two weeks, while an offer to be included in this study was made to all the HIV patient from several different hospitals. 27 patients responded affirmatively and a written informed consent form was obtained from all patients. The Ethics Committee of the participating hospitals approved the study. Inclusion criteria for HIV-infected patients on highly active antiretroviral therapy (HAART) interruption were age > 18 years, stable HAART, CD4^+ ^> 600/μL, and HIV-RNA load < 50 copies/ml for at least 6 months before discontinuing therapy. Patients were excluded if they previously had AIDS, ART failure, or poor adherence to HAART (< 90%), or if they were pregnant or had been splenectomized. Patients were evaluated monthly during the first three months and every two months thereafter. The criteria for reintroducing HAART, if needed, were: (i) patient's choice, at any CD4^+ ^count level; (ii) appearance of symptoms that could be manifestations of HIV infection (CDC category B events and/or AIDS defining events); and (iii) a decreasing CD4^+ ^count before falling below 350 cells/μL. We checked clinical parameters and measured T-cell subsets, HIV-RNA load, and lymphoproliferative responses (LPR) to different stimuli *in vitro*.

### T-cell subsets and HIV-RNA viral load

T lymphocyte CD4 cell counts were determined in whole blood by flow cytometry (FACScan, Becton-Dickinson Immunocytometry Systems, San Jose, CA, USA). Plasma HIV-1 RNA was quantified using the Amplicor Ultransensitive Assay (Roche Amplicor HIV-1 Monitor assay, version 1.5) with a limit of detection of 50 copies/mL.

### Lymphocyte proliferative assays

Peripheral blood mononuclear cells (PBMC) were seeded in 96-well flat-bottom microtiter plates (2 × 10^5^/200μl per well) resuspended in RPMI-1640 medium supplemented with 10% fetal calf serum. The cells were stimulated with several stimuli (p24 HIV-1 antigen (p24-Ag), streptokinase (SK), tetanus toxoid (TT), *Mycobacterium tuberculosis *purified protein derivative (PPD), and pokeweed). Cultures were made as reported previously [7]. The cells were harvested in glass fiber filters with an automatic cell harvester and radioactive incorporation was measured with a liquid scintillation spectrometer. Assays were carried out in quadruplicate and the results were expressed as "c.p.m. net": LPR of stimulated PBMC was corrected by subtracting the values of unstimulated PBMC.

### Multiplex cytokine analysis

Multiplex suspension bead array immunoassay was performed using the Luminex 100™ analyzer (Luminex Corporation, Austin, TX, USA) to identify protein expression in culture supernatants according to the manufacturers' specifications. Multiplex kit (LINCOplex™; LINCO Research, St. Charles, MO 63304, USA) was used to specifically evaluate IL-4 and INF-γ. A minimum of 100 events (beads) was collected for each of the analyte protein and median fluorescence intensities (MFI) were obtained. Analyte protein concentrations were automatically calculated based on standard curve data using MasterPlex™ QT Analysis version 2 (MiraiBio, Inc., Alameda, CA). A five-parameter regression formula was used to calculate the sample concentrations from the standard curves.

### Statistical analysis

All p-values were 2-tailed, and the threshold of significance was set at 0.05. All statistical analyses were performed with SPSS 12.0 software (SPSS INC, Chicago, IL, USA).

The outcome variables examined were analyzed by the Kaplan-Meier method. This was considered as the duration between the baseline timepoint (HAART interruption) and the appearance of a decrease in CD4^+ ^(values of CD4^+ ^< 350/μL, CD4^+ ^< 400/μL, and CD4^+ ^< 500/μL) and an increase of VL (values of VL > 10,000 copies/mL and VL > 30,000 copies/mL).

The value of immunological characteristics to predict declines in CD4^+ ^cell counts to < 350/μL in HIV-infected patients after treatment interruption was analyzed by Cox regression Immunologic characteristics (predictive markers) as a continuous and dichotomic variable. We did not make a multivariate analysis due to the low number of patients included in this study.

## Results

Table 1 shows the demographic, immunological and virological characteristics of the 27 HIV-infected patients who were included in the study. During follow up, one patient developed an acute retroviral syndrome and two patients presented thrombocytopenia (< 50,000 platelet/mm^3^) for which they had to restart HAART (with CD4+ > 350 cell/μL). The rest of patients remained completely asymptomatic and did not show signs of clinical progression.

**Table 1:** Clinical, immunological and virological parameters of 27 HIV-1-infected patients who discontinued antiretroviral therapy.

**Characteristic**	**Values**
N	27
Males	19 (70%)
Age in years	36.7 ± 1.09 (27; 52)
HCV coinfection	6 (22%)
HIV transmission category	
Intravenous drug user	4 (15%)
Homosexual	11 (41%)
Heterosexual	12 (44%)
Duration of HIV-infection in years	6.9 ± 0.6 (2; 14)
Symptomatic HIV disease, non AIDS	4 (16%)
Time on HAART in months	46.6 ± 3.1 (20.7; 95.1)
HIV-RNA, log_10 _copies/mL	4.2 ± 0.2 (2.3; 5.6)
Maximal HIV-RNA	4.4 ± 0.2 (2.7; 6.2)
Time with HIV-RNA < 50 inmonths	29.3 ± 2.3 (6; 56.2)
CD4^+ ^T-cell count/μL	
CD4^+ ^T-cell at HAART initiation	504 ± 50 (50; 1150)
% CD4^+ ^T-cell at HAART initiation	25.2 ± 1.4 (13; 38)
CD4^+ ^T-cell nadir	440 ± 42 (50; 1113)
CD4^+ ^T-cell at study entry, baseline	959 ± 55 (611; 1973)
C%D4^+ ^T-cell at study entry, baseline	39.1 ± 1.5 (28; 56)
CD4^+ ^T-cell increase on HAART	486 ± 60 (116; 1397)
%CD4^+ ^T-cell increase on HAART	14.6 ± 1.5 (0; 22)

Values are expressed as mean ± standard error mean (min.; max.) and absolute count.

### Evolution of immunological and virological markers

**Figure 1:**
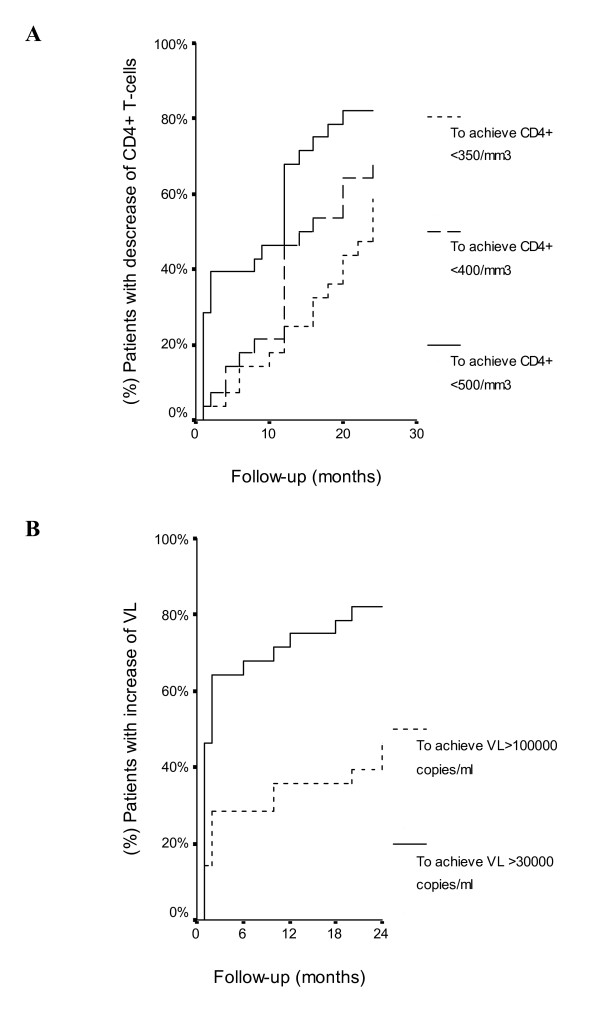
Kaplan-Meier for decreases in CD4+ T-cells (A) and rebound of viral load (VL) (B) in HIV-infected patients after treatment interruption.

Figure 1A shows the proportion of HIV-1-infected patients who experienced a decrease in CD4^+ ^24 months after treatment interruption. A decrease in the CD4^+ ^count was observed in all the patients, 23 (85%) patients experienced declines in CD4^+ ^cell count to < 500/μL, 19 (70%) in CD4^+ ^to < 400/μL, and 16 (59%) in CD4^+ ^to < 350/μL. The patients who experienced declines in CD4^+ ^cell count < 350/μL restarted HAART. Figure 1B shows details on the proportion of HIV-1-infected patients who experienced an increase in VL during follow-up. An increase in the VL was observed in all the patients. Twenty two (81%) patients had VL > 30,000 copies/mL and 13 (48%) patients had VL > 100,000 copies/mL. Interestingly, the patients with high HIV-RNA load when starting HAART had a high likelihood to achieve a HIV-RNA load rebound of both > 30,000 copies/mL (RR: 1.85 per log10; CI95%: 1.02; 3.35; p = 0.04) and > 100,000 copies/mL (2.45; 1.12; 5.36; p = 0.02) after ART interruption.

### Immunologic characteristics to predict declines in CD4^+ ^cell count < 350/μL

The analysis of characteristics shown in Table 2 concluded that the CD4^+ ^nadir, the CD4^+ ^count at of initiation of HAART, and the CD4^+ ^at study entry (at baseline) were all predictive of declines in CD4^+ ^cell count to < 350/μL during ART interruption in the period of study. We looked for functional characteristics of CD4^+ ^T cells that could be associated with the decline of CD4^+ ^count after ART discontinuation. Interestingly, lymphoproliferative responses to PPD at baseline was inversely related to a decline in CD4^+ ^cell count to < 350/μL during follow-up after ART interruption (Table 2). Patients with a LPR to PPD < 10,000 c.p.m. net at baseline had a relative risk (RR) of about 4.7 to experience a decline in CD4^+ ^cell count to < 350/μL. The analysis of LPRs to other stimuli (p24-Ag, SK, and TT) did not reveal any significant association.

**Table 2:** Summary of predictive value of immunologic characteristics for declines in CD4^+ ^cell counts to < 350/μL in HIV-infected patients after treatment interruption.

**Predictive markers**	**RR (CI95%)**	**p-values**
**CD4**^+^**(cells/μL) nadir**	0.99 (0.99; 1.01)	0.203
CD4^+ ^(cells/μL) nadir < 200/μL	3.37 (1.07; 10.36)	**0.038**
**CD4**^+^**(cells/μL) at starting HAART**	0.99 (0.99; 1.01)	0.192
CD4^+ ^(cells/μL) at starting HAART < 500/μL	2.39 (0.86; 6.61)	0.092
**CD4**^+^**(cells/μL) baseline**	0.99 (0.99; 1.00)	**0.015**
CD4^+ ^(cells/μL) at baseline < 750/μL	3.8 (1.28; 11.25)	**0.016**
**LPR to PPD (per 1000 c.p.m.) baseline**	0.95 (0.91; 1.00)	**0.050**
LPR to PPD < 10000 c.p.m.	4.77 (1.07; 21.12)	**0.039**
**IL-4 with PPD stimulus (per 100 pg/mL) baseline**	1.41 (1.01; 1.98)	**0.045**
IL-4 with PPD stimulus > 100 pg/mL	5.95 (1.76; 20.07)	**0.004**
**IL-4 with p24 antigen stimulus (per 100 pg/mL) baseline**	1.25 (1.01; 1.55)	**0.039**
IL-4 with p24 antigen stimulus > 100 pg/mL	3.14 (0.97; 10.15)	0.056

LPR: Lymphocyte proliferation. c.p.m.: count per minute. PPD: tuberculin purified protein derivative.

At starting HAART: when patients started their first highly active antiretroviral treatment.

At the entry of study (baseline): when patients stopped their antiretroviral treatment.

Similarly, IL-4 production in supernatant of PBMC cultured with PPD at baseline was also associated with an increased risk of experiencing a decline in CD4^+ ^cell count to < 350/μL during ART interruption (Table 2). Patients with IL-4 production > 100 pg/mL in supernatant of PBMC cultured with PPD at baseline had a RR of about 6 to experience a CD4^+ ^cell count decline to < 350/μL, and IL-4 production in supernatant of cultured PBMC with p24-Ag at baseline had a RR of about 1.2 to experience the same decline (Table 2).

## Discussion

This study confirms the importance of the CD4^+ ^count before initiation of antiretroviral therapy as a predictor of the need to reassume therapy during the 24 months following interruption of ART [8,9]. Not surprisingly, we have found that other immunological factors are predictors of the CD4^+^decrease after treatment interruption. Not only the number (CD4^+ ^nadir count) but also the function (lymphoproliferative response to some antigens) of the immune system seem to be important predictors of the CD4^+ ^decrease associated to discontinuation of ART in well controlled subjects.

As to be expected from previous reports, patients in this study experienced rebounds in HIV-RNA load to pre-HAART levels during TI [10]. Moreover, no immunological markers were associated with an increase of HIV-RNA load. We only found that the HIV-RNA load upon starting HAART had a positive association with HIV-RNA load rebounds of > 30,000 copies/mL and > 100,000 copies/mL.

HIV-1 infection is characterized by persistent viremia and a progressive decline in both the number and function of CD4^+ ^T cells, including the decrease of LPR to mitogens and antigens [11] and the lack of an effective HIV-1-specific immune response [12]. It is also clear that some patients have a greater impairment of the immune system than others during the course of HIV infection. HAART may cause the recovery of pathogen-specific immune responses in a majority of subjects, but HIV-specific immunity remains largely ineffective and most people with chronic HIV infection cannot control viremia after interruption of HAART [8,13]. In our study we found that the patients had a decrease in CD4^+ ^and increase of more than 4 log_10 _of HIV-RNA load during follow-up.

LPR *in vitro *is a good test to measure the quality of immune response. LPR to PPD is a marker of cellular immune damage, and its loss is associated with immunodeficiency [14]. The patients with a low LPR to PPD at baseline had a higher risk of their CD4^+ ^falling below 350/μL. This may be a consequence of prior severe immune damage leading to the loss of antigen-specific memory T cells [15]. So, LPR to PPD adds important quality information with regard to reaching low CD4^+ ^count in HIV-infected patients during ART interruption. Our results indicate that LPR to PPD appears to be a useful marker to measure the quality of immune response along with the quantification of CD4^+ ^(quantitative immune response) for the selection of patients to discontinue HAART. However, further study is required to confirm this data.

We also found that IL-4 production in supernatant of PBMC cultured with PPD and p24-Ag were strong predictive markers of CD4^+ ^falling below 350/μL. A previous report showed that there are two types of lymphocytes according to the cytokine secretion profile, and these profiles can be affected by HIV-infection [16]. Type-1 cells produce high levels of IFN-γ, IL-2, and TNF-α, while Type-2 cells preferentially secrete IL-4, IL-5 and IL-10 [16]. In addition to being secreted by CD4^+ ^T cells, it is now well known that some of these cytokines are also secreted by CD8^+ ^and non-T cells. It is documented that the type-1 cellular immune response is impaired during the course of HIV infection [17]. In our study, the type-2 cytokine IL-4 was quantified and a higher production of IL-4 in PBMC stimulated with PPD and p24-Ag was found. A possible explanation might be that an IL-4 increase produces a negative effect on IFN-γ production [16]. Thus, type-2 cells may increase type-2 responses (increased production of IL-4), B cells and/or suppress type-1 immune responses, thus compromising host response to infectious agents such as HIV [18].

The main limitation in our study is the small number of patients. Thus, while LPR to PPD may have been predictive of a certain outcome in this small number of patients it is premature to conclude that it is clinically useful for the selection of patients to discontinue HAART. We have not shown it to be predictive in a multivariate analysis. It is, in fact, quite possible that those individuals with lower CD4^+ ^nadirs are also those with poor CD4^+ ^T cell function and these assays simply identify the same individuals. Further studies to look at this as an independent predictor should be carried out with a larger number of patients. In spite of this, we consider our data to be very interesting as well as never having been previously described in non-structured treatment interruption.

## Conclusion

The conclusions of the study may be limited by the small number of patients evaluated. However, the results suggest that ART interruptions may be useful in a specific subset of patients with a minimal CD4^+ ^nadir and a good LPR response. Most significantly, we have identified immunological markers that may help explain the dynamics of CD4^+ ^count decline after discontinuing ART and may help identify patients that are most likely to benefit from a potential interruption of therapy.

## Competing interests

The author(s) declare that they have no competing interests.

## Authors' contributions

ES carried out the immunoassays and participated in the design of the study and performed the statistical analysis. SR participated in the design of the study, performed the statistical analysis, and contributed to the writing of the manuscript. SM, JCL, AM, RR, JGG, JRA, and FP carried out patient screening, collecting and recording data, and contributed to the writing of the manuscript. MAMF conceived of the study, and participated in its design and coordination. All authors read and approved the final manuscript.

## Pre-publication history

The pre-publication history for this paper can be accessed here:


